# Development of autoimmune thyroid disease after COVID-19 infection: case report

**DOI:** 10.3389/fmed.2024.1303855

**Published:** 2024-02-07

**Authors:** Si-na Du, Jian-wei Chen, Wei Li, Meng-chuan Wang, Yu-shan Mao

**Affiliations:** ^1^Department of Endocrinology, Cixi People Hospital Medical Health Group (Cixi People Hospital), Cixi, China; ^2^Department of Endocrinology, The Affiliated Lihuili Hospital of Ningbo University, Ningbo, China; ^3^Department of Endocrinology, The first Affiliated Hospital of Ningbo University, Ningbo, China

**Keywords:** SARS-CoV-2, Graves’ disease, autoimmune thyroiditis, hyperthyroidism, hypothyroidism

## Abstract

**Background:**

SARS-CoV-2 could trigger multiple immune responses, leading to several autoimmune diseases, including thyroid diseases. Many cases of thyroid diseases caused by COVID-19 infection have been reported. Here, we describe the disease development of patients with autoimmune thyroid disease after COVID-19 infection.

**Methods:**

The clinical characteristics, diagnosis and treatment of five different patients with autoimmune thyroid disease after COVID-19 infection were reported.

**Results:**

Female patients with primary autoimmune thyroid disease which have been stable for many years were reported. One month after COVID-19 infection, the disease has undergone different evolution. Case 1, a patient with history of long-term stable Hashimoto’s thyroiditis, suddenly suffered from Graves disease after COVID-19 infection. Case 2, a patient with history of long-term stable Hashimoto’s thyroiditis with thyroid nodules, suddenly suffered from Graves disease after infection. Case 3, a patient with history of long-term stable Graves disease, suddenly suffered from worsening after infection. The above three cases showed thyroid-stimulating antibodies were enhanced. Case 4, a patient with history of previous hypothyroidism had an increase in thyroid-related antibody (TPOAb and TRAb) activity after infection, followed by a marked worsening of hypothyroidism. Case 5, a patient with no history of thyroid disease suddenly developed controllable “thyrotoxicosis” after infection, suggesting the diagnosis of painless thyroiditis.

**Conclusion:**

The five case reports show a different development of the primary autoimmune thyroid disease after COVID-19 infection. The change in the trend of thyroid disease is closely related to the immune response induced by SARS-CoV-2 infection.

## Introduction

Since the end of 2022, with the change of COVID-19 epidemic prevention strategy in China, the number of infected individuals has significantly increased. Although respiratory symptoms are the most prominent clinical manifestation of patients with COVID-19, the involvement of other organs cannot be ignored (1). Since March 2020, the cases of thyroid disease related to COVID-19 infection have been reported successively (2). However, most studies focus on thyroid diseases caused by COVID-19 infection or SARS-CoV-2 vaccination (3). Limited information is available regarding potential changes in thyroid function after COVID-19 infection among individuals with a history of thyroid disease.

In this paper, we present a comprehensive analysis of 5 cases highlighting remarkable changes in the progression of thyroid disease after COVID-19 infection. These cases include the transition from Hashimoto’s thyroiditis with normal thyroid function to sudden Graves disease, the transition of Hashimoto’s thyroiditis and concurrent thyroid nodules with normal thyroid function to sudden Graves disease, the sudden exacerbation of pre-existing Graves disease, the pronounced deterioration of hypothyroidism, and the emergence of painless thyroiditis in previously normal individuals. We have carefully described the diagnosis and treatment measures for each case, provided valuable information for clinical management strategies for patients with a history of thyroid disease after COVID-19 infection. We aim to explore the mechanism between COVID-19 infection and thyroid pathophysiology, and ultimately promote individual diagnosis and treatment strategies.

## Case presentation

### Case 1

**Figure 1:**
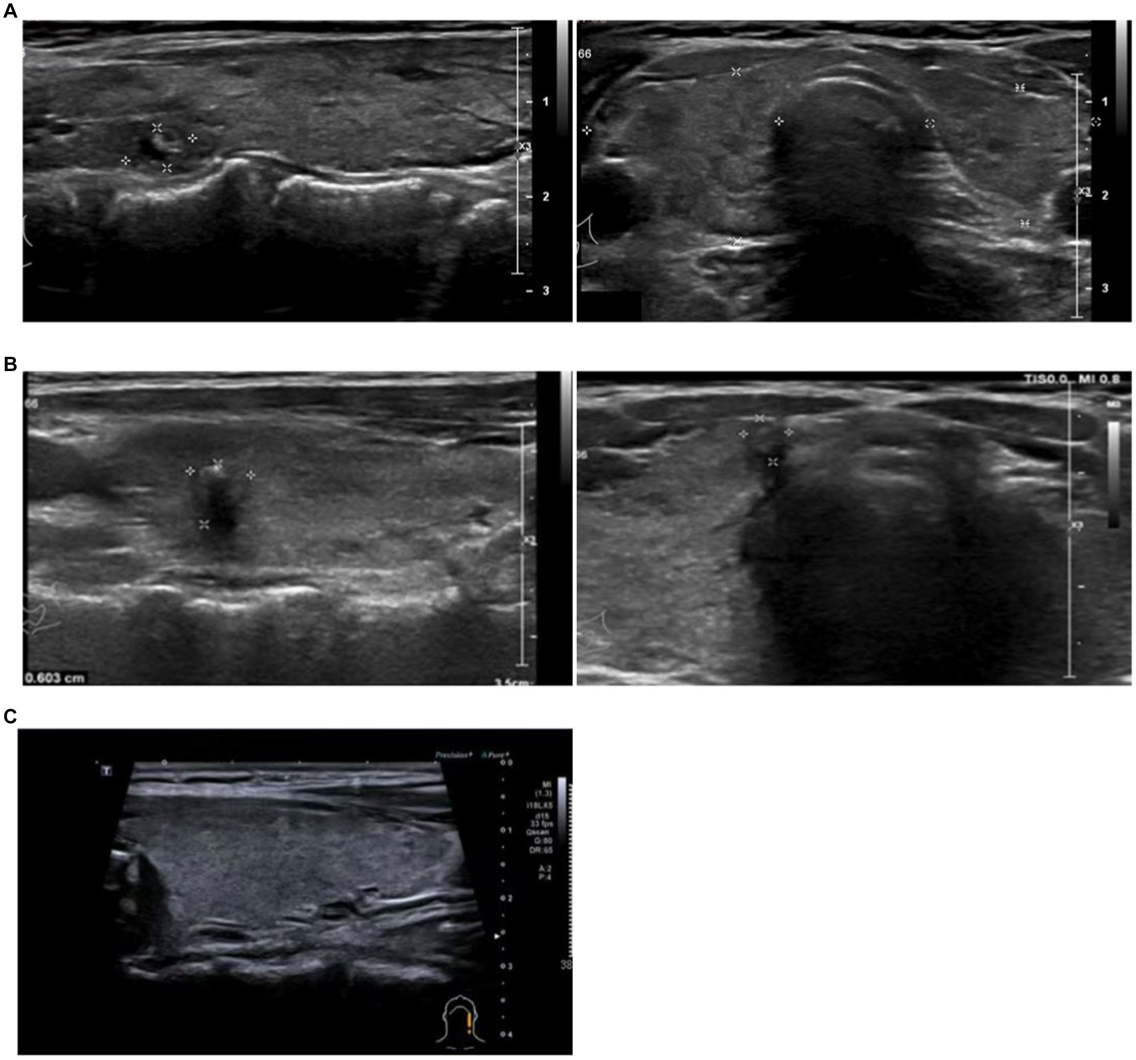
Thyroid ultrasonography. Thyroid ultrasonography of case 1 **(A)**, Right lobe: 20 × 13 mm. Left lobe: 17 × 9 mm. Bilateral thyroid echo thickening and bilateral thyroid nodules (label). Case 2 **(B)**, Right lobe: 20 × 20 mm. Left lobe: 21 × 19 mm. Bilateral thyroid echo thickening and bilateral thyroid nodules (label). Case 5 **(C)**, bilateral thyroid echo thickening.

The patient is a 53-year-old female with a previous history of Hashimoto’s thyroiditis. Her thyroid function has been in a normal state for 4 years without medication. She went to a doctor for “palpitation and emaciation for 1 month” after COVID-19 infection. Before the first episode of COVID-19, she had received two doses of the COVID-19 vaccine. Physical examination showed that there was no tenderness in the thyroid gland. Thyroid ultrasonography showed bilateral thyroid echo thickening and bilateral thyroid nodules (Figure 1A). Thyroid-related tests showed thyroid function indicated a significant increase in serum total triiodothyronine (TT3), total thyroxine (TT4), free triiodothyronine (FT3), and free thyroxine (FT4), while thyroid stimulating hormone (TSH) decreased, Anti-thyroid peroxidase antibody (TPOAb) significantly increased, and thyroid globulin antibody (TgAb) was positive. The TSH receptor antibody (TRAb) significantly increased (Table 1). The diagnosis was Graves disease. After 2 months of treatment with methimazole (Saizhi), the thyroid function returned to normal, and the treatment was uninterrupted. Up to July 2023, the thyroid function still remained within normal range. This case shows that a patient with long-term stable Hashimoto’s thyroiditis suddenly appeared hyperthyroidism after COVID-19 infection.

**Table 1:** Laboratory test results in the 5 included patients.

Cases	Date	TT3 (0.92–2.79 nmol/L)	TT4 (58–146 nmol/L)	FT3 (3.5–6.5 pmol/L)	FT4 (11-23 pmol/L)	TSH (0.5–4.8mIU/L)	TRAb (<1.5 IU/L)	TPOAb (0-9 IU/mL)	TgAb (0-4 IU/mL)	Therapy
Case 1	2019.3.27			5.38	15.28	1.771				Methimazole (Saizhi) 10 mg qd for 2 months
	2022.8.1	1.5	113.7	4.98	16.35	0.755		1,621	97.2	
	2023.3.24	6.04	369.9	26.67	68.53	<0.008	3.18	3477.6	73.4	
	2023.5.14	1.86	92.9	4.98	13.08	<0.008				
	2023.7.21	2.15	120.70	6.02	17.67	0.017				
Case 2	2022.8.24	2.33	157.2	5.56	19.58	0.707		837.2	72.2	Methimazole (Saizhi) 10 mg qd for 1 months
	2023.2.20	4.65	289.72	11.01	38.91	0.00			1.3	
	2023.3.27	5.70	295.80	19.75	45.05	<0.008		274		
	2023.4.5	3.14	150.25	7.49	17.82	0.01				
	2023.5.30	1.78	95.24	5.11	11.11	0.01		314	1.2	
	2023.6.16	1.67	90.85	4.66	8.79	1.45		223.50	0.70	
	2023.6.27						18.05			
Case 3	2021.3.28			4.47	8.83	12.697				Methimazole (Saizhi) 10 mg qd for 2 months
	2021.4.22			5.38	17.77	0.112				
	2021.4.25	1.21	117.99	5.14	16	0.1				
	2021.5.24			5.20	14.32	0.037	1.32	31.4	1.4	
	2021.6.12			5.00	14.50	0.025	1.12			
	2021.7.17			4.23	12.86	0.046		183.2	11.3	
	2021.8.19	135	96.78	3.92	12.75	0.812				
	2022.2.27	2.09	140.39	4.86	10.98	1.764				
	2022.5.7	2.12	127.04	4.03	10.09	1.704				
	2023.3.12	4.25	244.78	15.18	38.11	0.01	34.47	645.2	318	
	2023.4.30	2.43	133.0	6.98	17.5	0.039				
	2023.6.17	1.68	90.60	4.94	12.76	0.489				
Case 4	2020.9.6	1.11	91.13	3.99	20.80	0.63		61.4	<0.9	Levothyroxine 50 μg qd for 2 months	
	2020.11.14	1.37	99.61	4.92	18.80	0.93		27.7	<0.9		
	2021.1.29	1.33	90.24	4.25	21.31	0.49		17.4	<0.9		
	2021.5.2	1.20	69.60	3.32	17.50	0.43		19.3	<0.9		
	2021.8.8	1.92	96.20	5.40	21.00	<0.01		212	219		
	2022.2.6	0.87	8.23	3.41	1.48	0.67		43	<0.9		
	2023.2.1	1.31	79.66	4.26	18.39	1.25		521.52	100.30		
	2023.3.12	0.81	30.94	2.83	12.24	79.44		614.22	309.84		
	2023.5.7	1.31	79.66	4.26	18.39	1.25		521.52	100.30		
	2023.6.26	1.41	87.50	3.40	17.00	1.07		85.90	34.70	
Case 5	2018.3.15			4.94	10.16	1.62				Methimazole (Saizhi) 10 mg qd for 1 months
	2023.2.14	2.27	211.86	2.27	33.49	0	<0.25	21	2.2	
	2023.3.2	1.4	158.61	6.95	19.47	0				
	2023.3.16	1.08	135.61	6.44	13.58	0.01				
	2023.3.22	10.8	114.05	4.38	9.55	0.13				
	2023.4.19	1.07	92.21	5.37	10.60	3.73				
	2023.5.22	1.03	97.80	5.04	10.97	3.6		56.2	2.2	

The test data highlighted in red show above-normal, while blue show below-normal.TT3, Total triiodothyronine; TT4, Serum total thyroxine; FT3, Free triiodothyronine; FT4, Free thyroxine; TSH, Thyroid stimulating hormone; TPOAb, Anti-thyroid peroxidase antibody; TgAb, Thyroglobulin antibody; TRAb, TSH receptor antibody.

### Case 2

The patient was a 30-year-old female with a history of Graves disease with thyroid nodules. One year ago, fine needle aspiration revealed a benign nodule due to a type 4a thyroid nodule, which was not treated surgically. Regular thyroid function tests have been performed and medication has not been taken. Before the first episode of COVID-19, she had received two doses of the COVID-19 vaccine. In February 2023, the patient went to the hospital due to “panic and shaking for half a month” after COVID-19 infection in January (Table 1). After heart rate control observation for 1 month, hyperthyroidism worsened. The recheck of thyroid B ultrasound still showed that Hashimoto’s thyroid gland was accompanied by nodules (Figure 1B). TRAB was significantly elevated, and TPOAb was strongly positive. The diagnosis was “Graves disease, thyroid nodules,” After 1 month of treatment with methimazole (Saizhi), the thyroid function returned to normal, but TPOAb was still strongly positive. This case showed that a patient with long-term stable Hashimoto’s thyroiditis with thyroid nodules suddenly appeared Graves disease after COVID-19 infection.

### Case 3

The patient was a 32-year-old female with a history of Graves disease. The treatment with 5 mg qd of methimazole (Saizhi) has regularly continued for 2 years, and thyroid function has remained stable. In March 2023, the patient went to the hospital due to “Palpitation and hand tremor for 2 years, aggravated for more than 1 month” after COVID-19 infection in January. Before the first episode of COVID-19, she had received two doses of the COVID-19 vaccine. The symptoms of hyperthyroidism aggravated during scheduled reexamination. TT3, TT4, FT3, and FT4 increased significantly, TSH decreased to 0.01, TRAB increased significantly, TPOAb and TgAb increased significantly, and hyperthyroidism aggravated significantly (Table 1). After 2 months of treatment with methimazole (Saizhi) to 10 mg qd, the condition improved. This case showed that a patient with long-term stable Graves disease suddenly appeared exacerbation after COVID-19 infection.

### Case 4

The patient was a 25-year-old female with a history of hypothyroidism. The treatment with 12.5 μg qd of levothyroxine has regularly continued for 3 years, and thyroid function has remained stable. It was found that TPOAb and TgAb suddenly increased significantly, and the thyroid function was still within the normal range after COVID-19 infection in January. Before the first episode of COVID-19, she had received two doses of the COVID-19 vaccine. In March 2023, when the thyroid function was rechecked, it was found that TT3, TT4, FT3, and FT4 significantly decreased, and TSH significantly increased, with the continued increase of TPOAb and TRAb (Table 1). After treatment with levothyroxine, TPOAb and TRAb significantly blocked, the thyroid function returned to normal immediately. This case showed that a patient with long-term stable hypothyroidism suddenly appeared exacerbation of hypothyroidism after COVID-19 infection.

### Case 5

The patient was a 37-year-old female with normal thyroid function. In February 2023, the patient went to the hospital due to “slight neck pain for 1 week” after COVID-19 infection in January. Before the first episode of COVID-19, she had received two doses of the COVID-19 vaccine. Thyroid ultrasonography showed bilateral thyroid echo thickening (Figure 1C). Physical examination showed that TT3, TT4, FT3, and FT4 increased, TSH decreased to 0, TRAB was negative, TPOAb slightly increased, and ESR was normal. Considering “Hyperthyroidism,” she was treated with methimazole (Saizhi) to 10 mg qd for 1 month, then TT3, FT3, and FT4 returned to normal, except that TT4 slightly still rose (Table 1). The diagnosis of hyperthyroidism could not be determined. Stop medication for observation and fellow up regularly every month until the end of May 2023. Except for a slight increase in TPOAb, other functions were normal. This case may be considered subacute thyroiditis or painless thyroiditis. Considering the increase and persistence of TPOAb, patients may develop new onset AITD after the initial stage of subacute thyroiditis. This case showed that a patient with normal thyroid function suddenly appeared painless thyroiditis after COVID-19 infection.

## Discussion

Nowadays, Autoimmune thyroid disease (AITD) caused by COVID-19 infection have recently been reported, such as Grave’s disease, subacute thyroiditis, Hashimoto’s thyroiditis, Non thyroid illness syndrome and some unexplained thyroid dysfunction (4). At present, Hashimoto’s thyroiditis has been found in COVID-19 patients, who recovered to normal after treatment with levothyroxine (5). Brancatella (6) reported the characteristics of the first case of subacute thyroiditis caused by COVID-19 infection. The patient was an 18-year-old female, who had fever, neck pain, and mandibular radiation pain 18 days after COVID-19 infection. After receiving glucocorticoid treatment, thyroid function and inflammatory indicators returned to normal within 40 days. NTIS has been found in severe COVID-19 patients. Patients exhibited complex changes in the thyroid hormone spectrum, including decreased serum T3 levels and slight decreased in TSH levels. As the condition progresses, T4 also exhibited abnormal (7). A retrospective cohort study included 149 patients with COVID-19, and the incidence rate of Non thyroid Illness syndrome was approximately 28% (8). A cohort study from three hospitals in London included 334 COVID-19 patients and 122 non-COVID-19 patients. Only 5% COVID-19 patients had normal thyroid function. Compared with non-COVID-19 patients, the COVID-19 patients had lower levels of TSH and FT4, especially in severe cases (9). Harris (10) reported the first case of COVID-19 accompanied by Grave’s disease. Subsequently, Blanco (11) and Mateu Salat (12) reported four COVID-19 cases with Grave’s disease. The above 5 COVID-19 patients with Grave’s disease were all treated with methimazole, and the condition of Grave’s disease was controlled. Mechanically, stress response is a triggering factor for Grave’s disease, and SARS-CoV-2 infection as a stress factor, can directly lead to the occurrence of Grave’s disease. In addition, due to a lack of comprehensive understanding of COVID-19 in the early stage, the fear associated with COVID-19 may also be a trigger for Grave’s disease. None of the patients in the above studies had a history of thyroid disease, which was caused by COVID-19 infection. Furthermore, we cannot ignore another possibility that AITD may contribute to the development of the infection. Fallahi et al. have found a higher prevalence of COVID-19 infection in patients with AITD, indicating that patients with AITD are more susceptible to SARS-CoV-2 infection (13). In this paper, we report five cases of female patients with autoimmune thyroid disease that have been stable for many years. The evolution of the above 5 cases was different, and the trend of thyroid disease development was greatly enhanced in this special stage after COVID-19 infection.

The interaction relationship and molecular mechanism between thyroid disease and COVID-19 infection is complex. At present, studies have showed that autoimmunity played a very important role (14). The target of SARS-CoV-2 injury may originate from the thyroid gland and the entire hypothalamic pituitary thyroid axis, and may manifest as thyroid toxicity and hypothyroidism. SARS-CoV-2 can directly damage thyroid tissue through transmembrane serine protease 2 and angiotensin converting enzyme 2 receptors on the surface of thyroid follicular cells, leading to thyroiditis like other respiratory viruses (15). Another mechanism is molecular mimicry. Antibodies targeting SARS-CoV-2 may cross react with their own tissue antigens, such as TPO. This antibody can interact with different tissue antigens, including thyroid tissue (16). In addition, infection or vaccination that causes innate immune stimulation or cytokine storms, ultimately leading to the stimulation of self reactive T cells, which is also considered the cause of thyroid damage (17–19). In COVID-19 cases with concomitant Non thyroid illness syndrome, inflammatory indicators such as CRP, erythrocyte sedimentation rate, and procalcitonin significantly increased, indicated a strong inflammatory and immune response (8). Neutrophil extracellular traps and neutrophil-related cytokines also play an important role (20). The study found that IL-6 and TNF-α significantly elevated in patients with COVID-19 infection and AITD. SARS-CoV-2 induced Th17 cell proliferation and differentiation through cytokines such as IL-6, induced the production of specific autoreactive B cells, and induced AITD or other thyroid diseases by disrupting immune tolerance (21, 22). Moreover, Researchers found that ACE2 and transmembrane serine protease 2 (TMPRSS2) are important receptors for SARS-CoV-2 to invade cells (23). ACE2 and TMPRSS2 are highly expressed in the thyroid (even higher than lung tissue). Virus S protein binds to TMPRSS2, causing tissue and organ damage through toxic effects on target cells and activation of immune cells (24, 25).

In terms of clinical treatment, there is no significant difference from conventional treatment, and the dosage of medicine should be adjusted as needed. The 5 cases in this article show stable condition after medication treatment. It is worth noting that in some patients with thyroid diseases aggravated by COVID-19 infection, the thyroid gland can partially repair itself, so the drug dose needs to be adjusted in time.

There are also several limitations. Firstly, beside SARS-CoV-2, the possibility of co-infection with other viruses that have been associated with AITD cannot be disregarded, even if SARS-CoV-2 was detected. Secondly, besides SARS-CoV-2, SARS-CoV-2 vaccines may also cause AITD. The role of adjuvants in vaccines as potential contributors to “adjuvant-induced autoimmune/inflammatory syndrome” in AITD remains a consideration that cannot be dismissed. Additionally, it is essential to acknowledge the inherent limitations of our case report, including the potential for excessive interpretation and selection bias. Therefore, without comprehensive and precise descriptions, the establishment of causal relationships remains elusive, necessitating a deeper exploration. Despite these limitations, the case report offers valuable insights into the progression of COVID-19, holding significant relevance for clinical practice. The increasing accumulation of clinical cases will help facilitate the elucidation of the mechanisms underpinning virus-induced AITD, ultimately enhancing diagnostic and therapeutic strategies.

## Conclusion

COVID-19 infection can induce some autoimmune diseases and aggravate the original AITD disease. AITD patients may have complex disease evolution after infection with COVID-19 due to the large number of patients and strong differences in clinical symptoms. Both doctors and patients need to be vigilant and adjust treatment plans promptly based on the condition.

## Data availability statement

The raw data supporting the conclusions of this article will be made available by the authors, without undue reservation.

## Ethics statement

The studies involving humans were approved by Ethics Committee of Cixi People's Hospital. The studies were conducted in accordance with the local legislation and institutional requirements. The participants provided their written informed consent to participate in this study. Written informed consent was obtained from the individual(s) for the publication of any potentially identifiable images or data included in this article.

## Author contributions

S-nD: Data curation, Investigation, Methodology, Supervision, Validation, Writing – original draft. J-wC: Data curation, Investigation, Supervision, Validation, Writing – review & editing. WL: Conceptualization, Data curation, Formal analysis, Investigation, Methodology, Writing – review & editing. M-cW: Data curation, Investigation, Writing – review & editing. Y-sM: Conceptualization, Data curation, Funding acquisition, Methodology, Project administration, Supervision, Validation, Writing – review & editing.
